# Reproducible bioinformatics project: a community for reproducible bioinformatics analysis pipelines

**DOI:** 10.1186/s12859-018-2296-x

**Published:** 2018-10-15

**Authors:** Neha Kulkarni, Luca Alessandrì, Riccardo Panero, Maddalena Arigoni, Martina Olivero, Giulio Ferrero, Francesca Cordero, Marco Beccuti, Raffaele A. Calogero

**Affiliations:** 1 Department of Molecular Biotechnology and Health Sciences, University of Torino, Torino, Italy; 2 Department of Oncology, University of Torino, Candiolo, Italy; 3 Department of Computer Sciences, University of Torino, Torino, Italy

**Keywords:** Reproducible research, Docker, Whole transcriptome sequencing, microRNA sequencing, Chromatin Immuno precipitation sequencing, Community, Single nucleotide variants

## Abstract

**Background:**

Reproducibility of a research is a key element in the modern science and it is mandatory for any industrial application. It represents the ability of replicating an experiment independently by the location and the operator. Therefore, a study can be considered reproducible only if all used data are available and the exploited computational analysis workflow is clearly described. However, today for reproducing a complex bioinformatics analysis, the raw data and the list of tools used in the workflow could be not enough to guarantee the reproducibility of the results obtained. Indeed, different releases of the same tools and/or of the system libraries (exploited by such tools) might lead to sneaky reproducibility issues.

**Results:**

To address this challenge, we established the *Reproducible Bioinformatics Project (RBP)*, which is a non-profit and open-source project, whose aim is to provide a schema and an infrastructure, based on docker images and R package, to provide reproducible results in Bioinformatics. One or more Docker images are then defined for a workflow (typically one for each task), while the workflow implementation is handled via R-functions embedded in a package available at github repository. Thus, a bioinformatician participating to the project has firstly to integrate her/his workflow modules into Docker image(s) exploiting an Ubuntu docker image developed ad hoc by RPB to make easier this task. Secondly, the workflow implementation must be realized in R according to an R-skeleton function made available by RPB to guarantee homogeneity and reusability among different RPB functions. Moreover she/he has to provide the R vignette explaining the package functionality together with an example dataset which can be used to improve the user confidence in the workflow utilization.

**Conclusions:**

Reproducible Bioinformatics Project provides a general schema and an infrastructure to distribute robust and reproducible workflows. Thus, it guarantees to final users the ability to repeat consistently any analysis independently by the used UNIX-like architecture.

## Background

Recently Baker and Lithgow [1, 2] highlighted the problem of the reproducibility in research. Reproducibility criticality affects to different extent a large portion of the science fields [1]. Since nowadays bioinformatics plays an important role in many biological and medical studies [3], a great effort must be put to make such computational analyses reproducible [4, 5]. Reproducibility issues in bioinformatics might be due to the short half-life of the bioinformatics software, the complexity of the pipelines, the uncontrolled effects induced by changes in the system libraries, the incompleteness or imprecision in workflow description, etc. To deal with reproducibility issues in Bioinformatics Sandve [5] suggested ten good practice rules for the development and the utilization of a computational workflow (Table 1). A community that fulfills some of the rules suggested by Sandve is Bioconductor [6] project, which provides version control for a large amount of genomics/bioinformatics packages. In this way, old releases of any Bioconductor package are kept available for the users. However, Bioconductor does not cover all the steps of any possible bioinformatics workflow, e.g. in RNAseq wolkflow fastq trimming and alignment steps are generally done using tools not implemented in Bioconductor. BaseSpace [7, 8] and Galaxy [9] represent an example of both commercial and open-source cloud solutions, which partially fulfill Sandve’s roles. Furthermore, the workflows implemented in such environments cannot be heavily customized, e.g. BaseSpace has strict rules for applications submission. Moreover, clouds applications have to cope with legal and ethical issues [10].

**Table 1:** Good practice bioinformatics rules, derived from Sandve et al. [5]

1	For Every Result, Keep Track of How It Was Produced
2	Avoid Manual Data Manipulation Steps
3	Archive the Exact Versions of All External Programs Used
4	Version Control All Custom Scripts
5	Record All Intermediate Results, When Possible in Standardized Formats
6	For Analyses That Include Randomness, Note Underlying Random Seeds
7	Always Store Raw Data behind Plots
8	Generate Hierarchical Analysis Output, Allowing Layers of Increasing Detail to Be Inspected
9	Connect Textual Statements to Underlying Results
10	Provide Public Access to Scripts, Runs, and Results

Galaxy instead implements the functional reproducibility level, i.e. the information about data and the utilized tools are saved in terms of meta-data, while RBP exploiting Docker framework provides also the computation reproducibility, i.e. the real image of the computation environment used to generate the date is stored.

Recently container technology, a lightweight Operation System (OS)-level virtualization, was explored in the area of Bioinformatics to make easier the distribution, the utilization and the maintenance of bioinformatics software [11–13]. Indeed, since applications and their dependencies are packaged together in the container image, the users have not to download and install all the dependencies required by an application, thus avoiding all the cases where the dependencies are not well documented or not available at all. Moreover, problems related to versions conflicts or updates of the system libraries do not occur, because the containers are isolated and frozen from the rest of the operating system.

Among the available container platforms, Docker (http://www.docker.com) is becoming de facto the standard environment to quickly compose, create, deploy, scale and oversee containerized applications under Linux. Its strengths are the high degree of portability, which allows users to register and share containers over various hosts in private and public repositories, and to achieve a more effective resource use and a faster deployment compared with other similar software.

In Menegidio [13], da Veiga [11] and Kim [12] the authors provide a large collection of bioinformatics tools containerized in a Docker image called BioContainers. However, a controlled and flexible framework to create and distribute bioinformatics reproducible workflow is not defined. Instead, projects like (https://snakemake.bitbucket.io) or Nextflow (https://www.nextflow.io) allow users to create reproducible and scalable data analyses specifying their own pipeline through a powerful metalanguage for workflow specification. However, the strong flexibility of these metalanguages can make difficult their utilization for users without advanced programming skills.

To cope with these aspects, we propose the implementation of the Reproducible Bioinformatics Project (RBP, http://reproducible-bioinformatics.org/), whose aims are (i) to distribute to the bioinformatics community docker-based applications under the reproducibility framework proposed by Sandve [5], and (ii) to provide to R bionformatics community an easier framework for the developing their own reproducible workflows.

The concept of BioContainers, described above, is different from RBP project. BioContainers provides pieces of software to be integrated in a workflow, as instead in RBP complete workflows are provided, e.g. gene/transcripts RNAseq, microRNA-sequencing (miRNA-seq), Chromatin Immuno Precipitation sequencing (ChIP-seq), DNA/RNAseq variant calling. RBP docker images not only include the specific software that give the name to the image, e.g. in bwa RBP docker image, bwa.2017.01, samtools, picard-tools, java and R, are also present.

RBP accepts simple docker implementations of *bioinformatics software* (e.g. a docker embedding bwa aligner tool), implementation of *complex pipelines* involving the use of multiple dockers images (e.g. a RNAseq workflow providing all the steps for an analysis starting from the quality control of the fastq to differential expression), as well as *demonstrative workflows (*i.e. docker images embedding the full bioinformatics workflow used in a publication) intended to provide the ability to reproduce published data.

## Methods

**Fig. 1:**
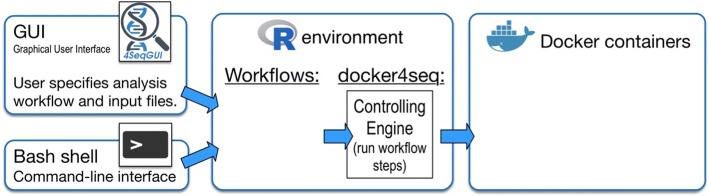
Reproducible Bioinformatics Project structure

The Reproducible Bioinformatics Project (RBP) reference web page is http://reproducible-bioinformatics.org. The project is based on three modules (Fig. 1): (i) *docker4seq R package* (https://github.com/kendomaniac/docker4seq), (ii) *dockers images* (https://hub.docker.com/u/repbioinfo/), and (iii) *4SeqGUI* (https://github.com/mbeccuti/4SeqGUI).

*Docker4seq* package provides the interface between users and docker containers. *Docker4seq* is organized in two branches: stable and development. The transition between development and stable branch is done when a module (R function(s)/docker container(s)) fulfills the 10 rules suggested by Sandve [5] for the good bioinformatics practice (Table 1).

The function *skeleton.R* in docker4seq provides a prototype to build a docker controlling function. A tutorial on how to use the skeleton.R function is available in the section “How to be part of the Reproducible Bioinformatics project” at http://www.reproducible-bioinformatics.org/ and the skeleton.R is part of the devel branch of docker4seq (https://github.com/kendomaniac/docker4seq/tree/devel). The tutorial also embeds a description of the Ubuntu docker image called via skeleton.R. In the docker images repository *docker.io/repbioinfo* is available an Ubuntu image, which is the starting image used for the creation of all docker images developed by the RBP core team. Since, there are no specific software requirements for the docker images present in RBP, developers can use any linux image to build their own docker image.

Acknowledgments of the developer work is provided within the structure of the *skeleton.R.* In *skeleton.R* there is a field indicating developer affiliation and email for contacts.

Developer is free to decide to use this prototype or to adapt a different Linux docker distribution for his/her application. Docker images designed by the core developers of RBP are located in *docker.io/repbioinfo* (docker.com), the images developed by third parties can be instead placed in any public-access docker repository.

RBP requires that any operation, implying the use of any R/Bioconductor packages or the use of an external software, has to be implemented in a docker container. Only reformatting actions, e.g. table assembly, data reordering, etc., can be handled outside a docker image.

Any new RBP module (R function(s)/docker image(s)) must be associated with an explanatory vignette, accessible online as html document, and with a set of test data accessible online. Thus, all instruments needed to acquire confidence on module functionalities are provided to the final user.

Docker images are labelled with the extension YYYY.NN, where YYYY is the year of insertion in the stable version and NN a progressive number. YYYY changes only if any update on the program(s), implemented in the docker image, is done. This because any of such updates will affect the reproducibility of the workflow. Previous version(s) will be also available in the repository. NN refers to changes in the docker image, which do not affect the reproducibility of the workflow.

A new module can be submitted to the info@reproducible-bioinformatics.org and RBP core team will verify the compliance with Sandve [5] rules. Specifically, to guarantee the compliance with Sandve rules, RBP core team will check that:

- Each new workflow produces for each analysis step a log file, thus tracking how the results are produced (Sandve rule 1).
- All workflow/module steps are executed through scripts, thus avoiding manual data manipulation steps (Sandve rule 2).
- All computation events are executed within a docker container and the versions of the software embedded in the docker image is shown as tag of the docker image (Sandve rule 3, 4).
- All intermediate results are available as part of the final results (Sandve rule 5).
- In case random seeds are used, they are recorded in a file and provided as part of final output of the module (Sandve rule 6).
- Raw data used to generate plots should be made available with plots (Sandve rule 7).
- Sandve rules 8 and 9 are not considered mandatory, because are mostly dependent from the workflow/module. The RBP core team will check if compliance to these rules will improve the overall quality of workflow/module output.
- License associated with the modules/workflows embedded in docker4seq must guarantee public access to the scripts and docker images (Sandve rule 10).

Rules 8 and 9, reported in Table 1, are not considered mandatory.

Ones validated, the R functions controlling the new module are inserted into *docker4seq* stable release. Partially validated modules will be placed in development branch and moved to stable one when compliance with Sandve’s rules is fulfilled.

4SeqGUI is a Java based graphical interface to docker4seq functions. It is designed to provide a GUI to users having limited knowledge of R scripting. Currently the GUI embeds only general-purpose workflows, such as RNAseq, miRNA-seq and Chip-seq workflow.

## Results

**Fig. 2:**
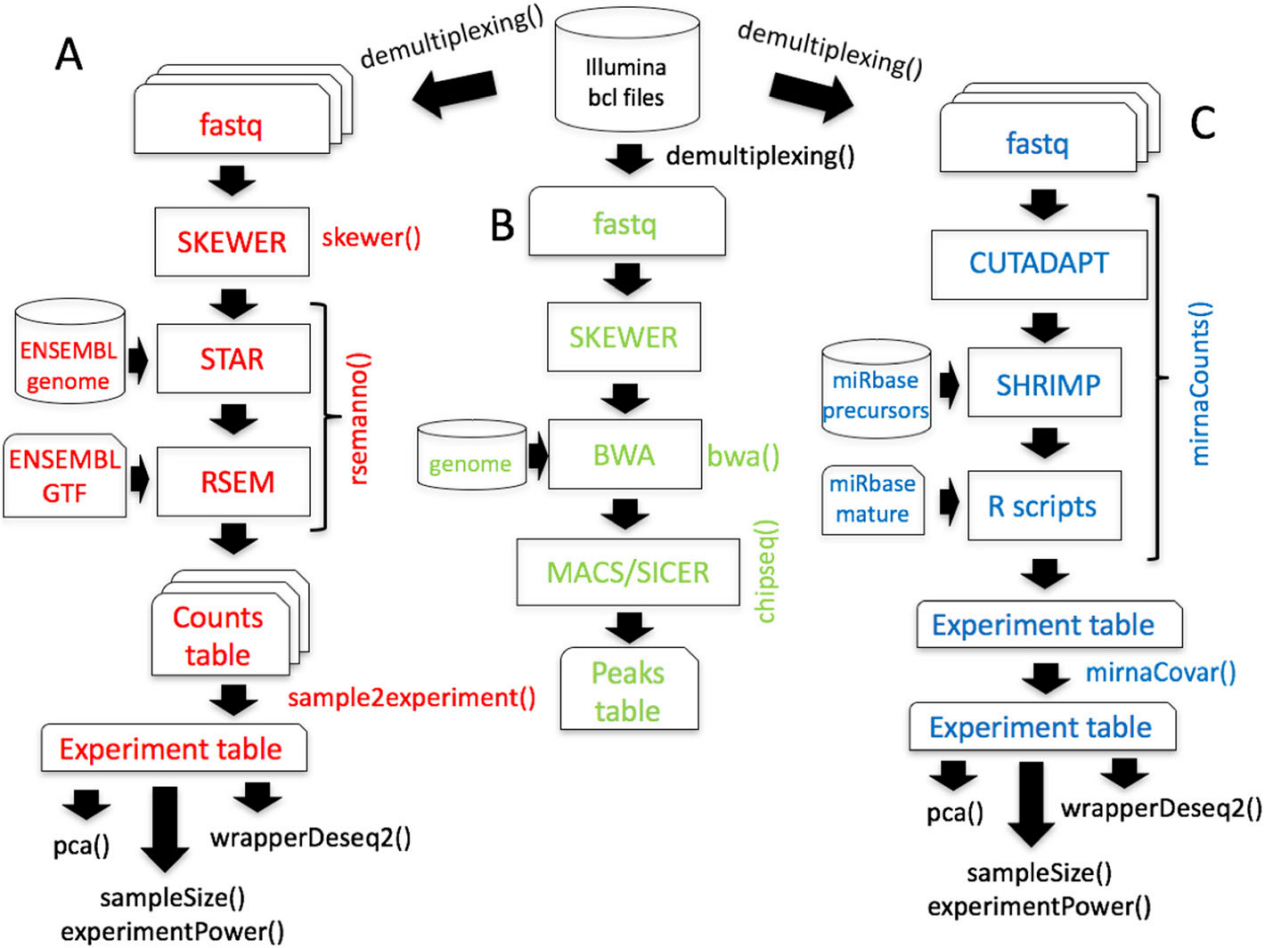
Workflows available in the stable branch of docker4seq. **a** Whole transcriptome sequencing workflow, **b** ChIP sequencing workflow, and **c** miRNA sequencing workflow. The names followed by parenthesis are the docker4seq functions used to execute the analysis steps. Black indicate elements in common among more than one workflow

The stable branch of *docker4seq R package* contains all the R functions required to handle all the steps of RNAseq workflow (Fig. 2a), ChIP-seq workflow (Fig. 2b), and miRNA-seq workflow (Fig. 2c). *Docker4seq* also provides a wrapper function for the *bcl2fastq* Illumina tool to convert the Illumina sequencer output in demultiplexed fastq files (Fig. 2). Then, the fastq files can be handled with any of the three different workflows. The counts table produced by RNAseq or miRNAseq workflows can be used to data visualization (*pca,* principal component analysis function), to evaluate the statistical power of the experiment (*experimentPower* function), to define the optimal sample size of the experiment for the detection of differentially expressed genes (*sampleSize* function) and to detect differentially expressed genes/transcripts (*wrapperDeseq2* function). Sample size/statistical power estimation of the experiment and differential expression are calculated respectively via RnaSeqSampleSize [14] and DESeq2 Bioconductor packages [15].

In the development branch, we work on three workflows (i) Patient Derived Xenograft (PDX) workflow, (ii) human small non-conding (snc) RNAs workflow, and (iii) B-cell clonality and Minimal Residual Disease detection.

**Fig. 3:**
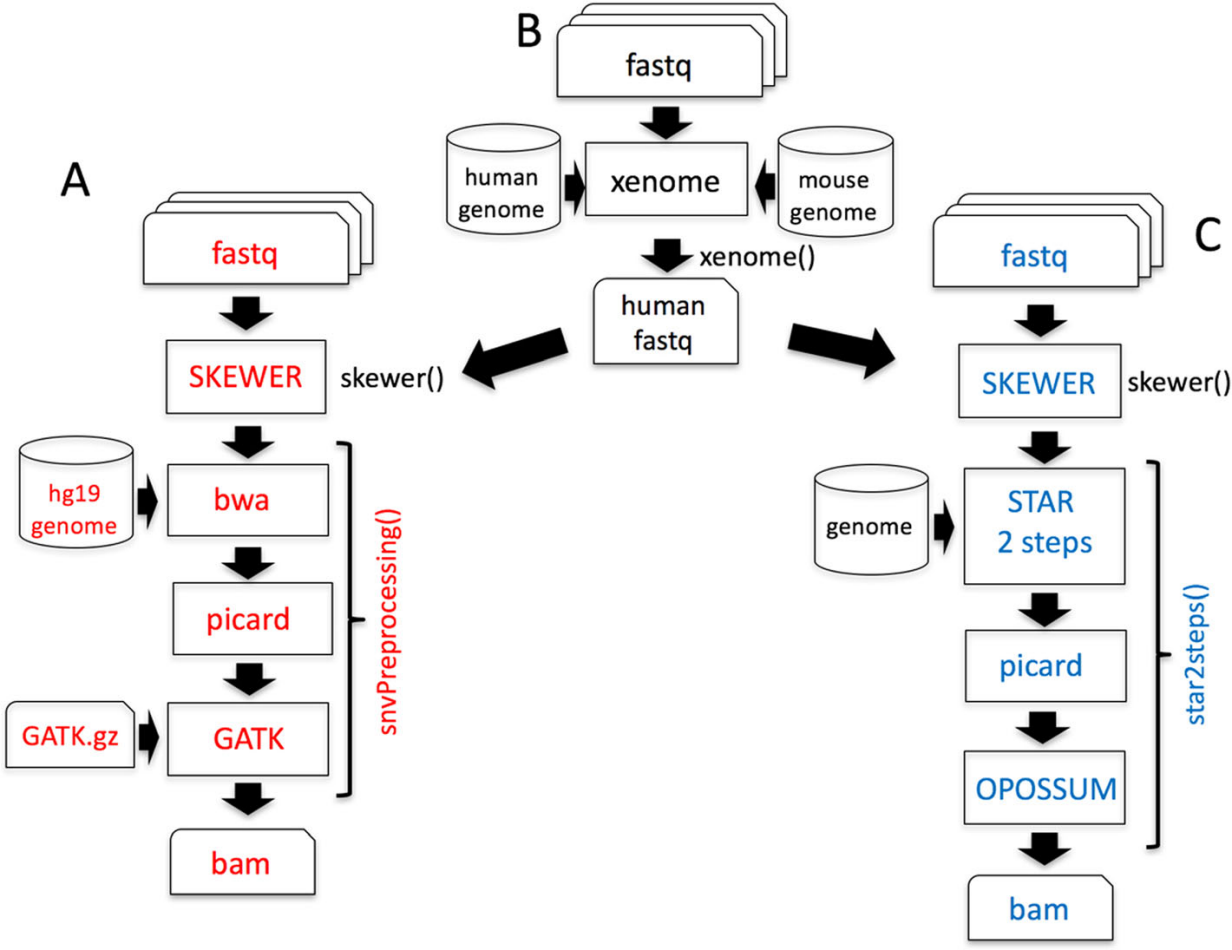
Variant calling workflows under refinement in the development branch of docker4seq. **a** SNVs calling in DNA workflow. The function *snvPreprocessing* requires that users provides its own copy of the GATK software, because of Broad Institute license restrictions. This function returns a bam file sorted, with duplicates marked after GATK indel realignment and quality recalibration. **b** Data preprocessing for samples derived by Patient Derived Xenografths (PDX). The *xenome* function discriminates between the mouse host reads and the human tumor reads, then DNA or RNA SNV calling workflows can be applied. **c** SNVs calling in RNA workflow. The function *star2steps* generates a sorted bam, where duplicates are marked and processed by opossum for removal of intronic regions and merging of overlapping reads. The names followed by parenthesis are the docker4seq functions used to execute the analysis steps. Black indicate elements in common between more than one workflow

**Fig. 4:**
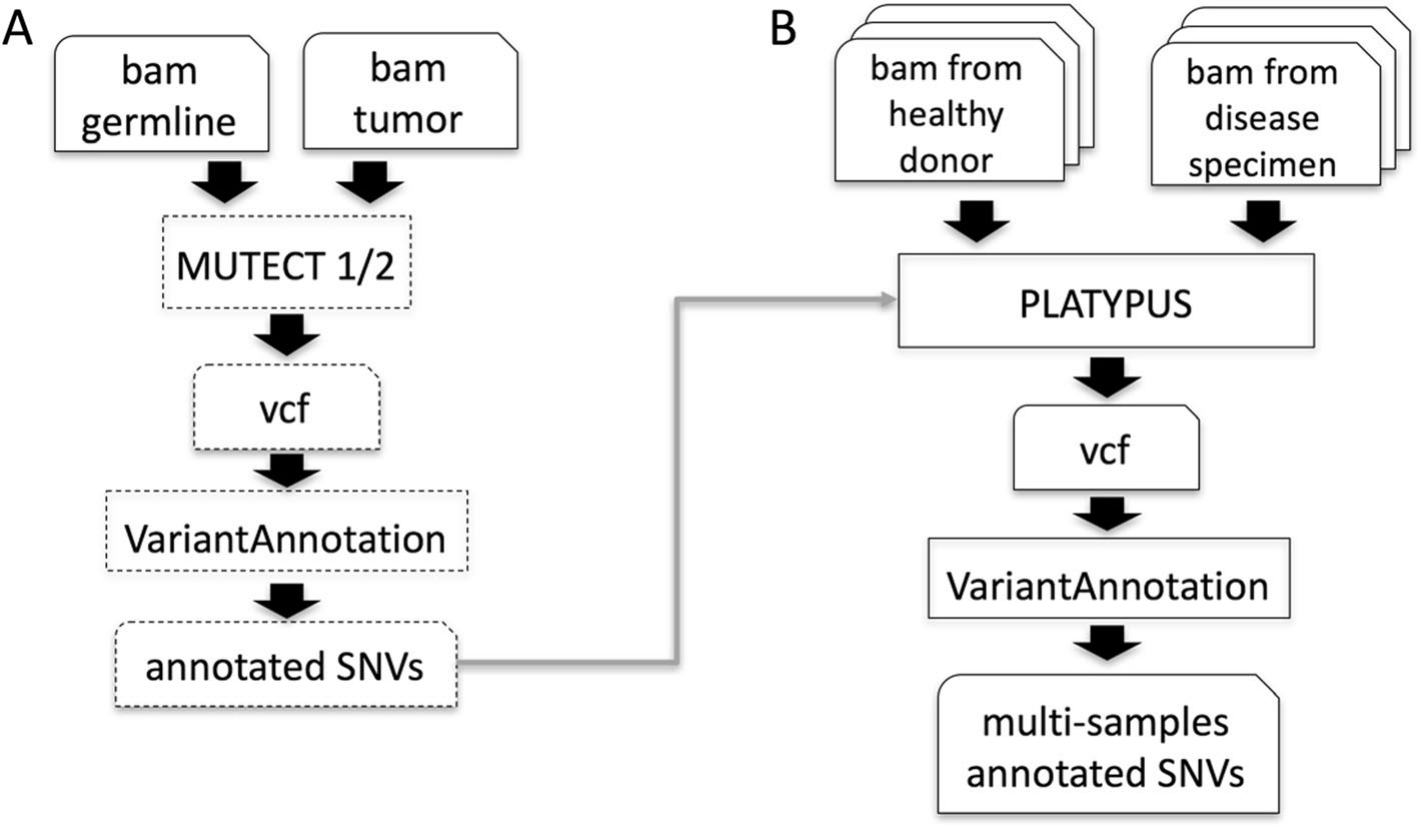
Variant calling workflows under development in the development branch of docker4seq. **a** Somatic SNVs detection using GATK MUTECT 1 or 2. **b** Platypus based join mutations caller. Dashed blocks are not implemented, yet

In the first workflow we provide a pipeline for DNA (from EXOMEseq data) and RNA (from RNAseq data) somatic variant calling. The DNA variant calling workflow embeds the pre-processing procedure suggested by the GATK best practice (Fig. 3a). RNAseq data preparation for variant calling (Fig. 3c) requires the use of STAR 2 step procedure [16], which provides significantly increased sensitivity to novel splice junctions. Then, after sorting and duplicates marking, OPOSSUM [17] is used to remove intronic regions and to merge overlapping reads. We have also implemented a specific procedure (Fig. 3b), based on xenome software [18], to discriminate between human reads and mouse host reads in the sequences produced by the analysis of patients derived xenografts (PDX, [19]). As part of the somatic variant calling workflow we are implementing MUTECT 1 and 2 [20] (Fig. 4a) to call somatic variants as well as PLATYPUS [21] for extracting information of joined-samples Single Nucleotide Variants (SNVs)(Fig. 4b).

We are also expanding the RNAseq module adding the reference-free Salmon aligner [22], which employs less memory for the alignment task than STAR, but providing similar results [23].

**Fig. 5:**
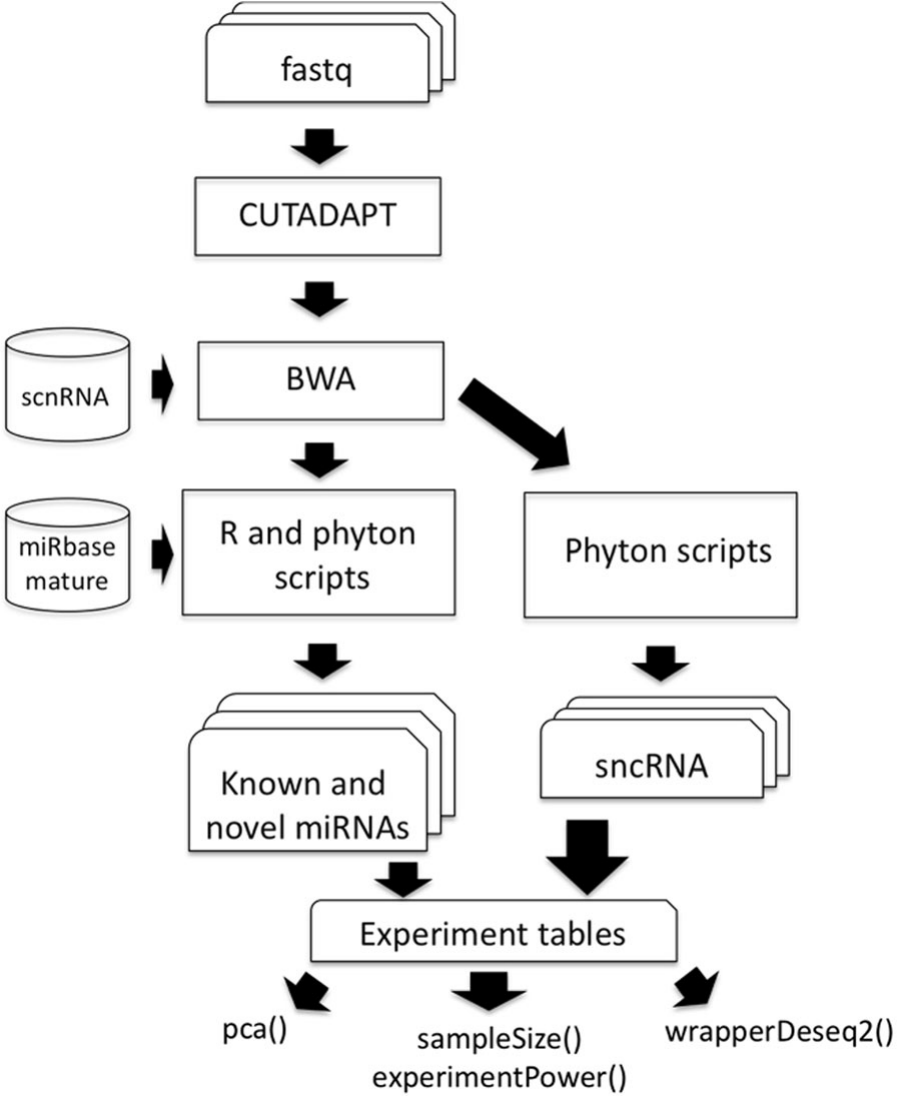
sncRNA workflow. The sncRNA pipeline starts from a reference composed by the set of sncRNAs that contains all sncRNA characterized by a length minor than 80 bp. Then, two types of scripts are used one dedicated to the detection of known and novel microRNAs while the other is focused on sncRNAs

The second workflow, used in the analysis described in the paper by Ferrero et al. [24], is focus on the analysis of sncRNAs as reported in Fig. 5. The quality of the FASTQ files are checked using FastQC software. The reads associated with good quality values are clipped from the adapter sequences using Cutadapt. The trimmed reads are then mapped against an in-house reference of human small RNA sequences composed of: (i) 1881 precursor miRNA sequences downloaded from miRBase (Release 21) (ii) 32,826 piRNA sequences from piRBase v1.0, and (iii) 5171 small RNA sequences from Database of Small Human non-coding RNAs (DASHR) database v 1.0 shorter than 80 bp.

The alignment is performed using the BWA algorithm. Small RNAs quantification is performed differently between miRNAs and non miRNAs sncRNAs. The miRNA expression is quantify using two methods, called annotation-based or the position-based method respectively. In the annotation-based method, mature miRNAs expression quantification is performed by counting the read mapped on miRBase mature miRNA sequences using an GenomicRanges R package. Since not all miRNA mature sequences are annotated in miRBase, the position-based read count method is performed by considering the read mapping position within the precursor miRNA sequences. The result of the two quantification methods are merged into a final miRNA count matrix. In this matrix each mature miRNA not annotated in miRBase but quantified using the position-based method is reported with suffix *Novel*. Quantification of non miRNA annotations is performed counting the read alignment reported by BWA output sam files. The identification of Differentially Expressed sncRNAs is performed using Deseq2 package as reported in the RNAseq workflow.

**Fig. 6:**
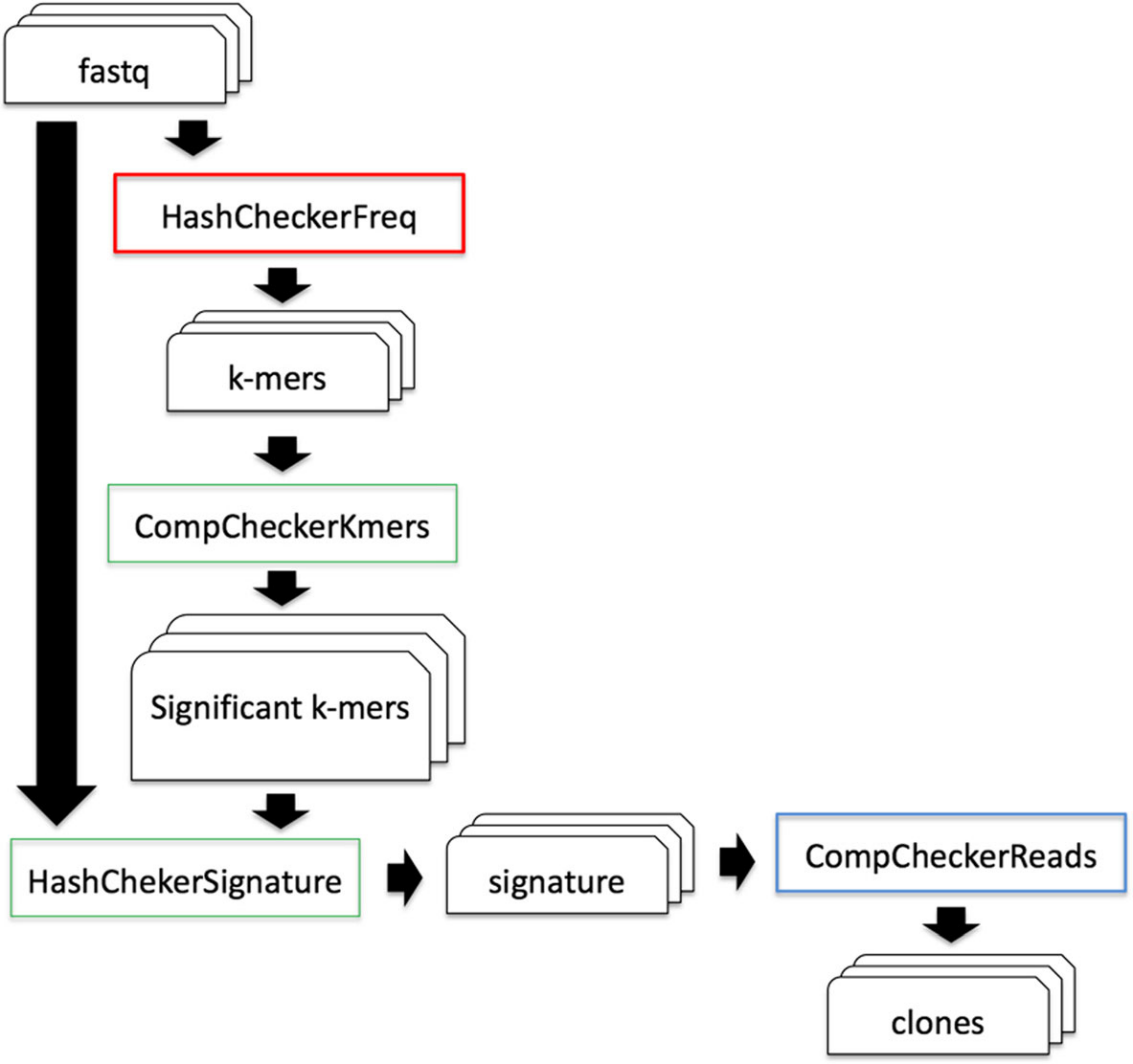
HashClone pipeline. The HashClone strategy is organized in three steps: The first step (red box) is used to detect k-mer in all patients’ samples. The second step (green box) focus on the generation of sequence signatures leading to the identification of the set of putative clones present in each of the patients’ sample; the third step (blue box) is used to the characterization and evaluation of the cancer clones

The third workflow is based on the HashClone framework [25, 26] a new suite of bioinformatics tools providing B-cells clonality assessment and minimal residual disease (MRD) monitoring over time from deep sequencing data, was integrated in the *Docker4seq* package. In particular, a parallel version of the standard HashClone workflow (Fig. 6) was developed exploiting the docker architecture.

All the modules described above are implemented in 22 docker images deposited in the docker hub (https://hub.docker.com/u/repbioinfo/).

As part of the RBP we have also developed a GUI, 4SeqGUI (https://github.com/mbeccuti/4SeqGUI). The GUI is implemented in JAVA and can be exploited to perform whole transcriptome sequencing workflow (Fig. 2a), ChIP sequencing workflow (Fig. 2b), and miRNA sequencing workflow (Fig. 2c).

## Discussion

RBP core developers created frameworks for RNA/miRNA quantification and analysis. ChIPseq workflow was also developed and variant calling workflows for DNA and RNA are under active development. A peculiar feature of RBP is the acceptance of *demonstrative workflows*, i.e. bioinformatics procedures described in a biological/medical paper. A demonstrative workflow is wrapped in a docker image and it is supported by a tutorial, which describes step by step how the analysis is done to guarantee the reproducibility of published data.

## Conclusions

Bioinformatics workflows are becoming an essential part of many research papers. However, absence of clear and well-defined rules on the code distribution make the results of most published researches unreproducible [27]. Recently, Almugbel and coworkers [28] described an interesting infrastructure to embed Bioconductor based packages. However, Bioconductor does not cover all steps of any possible bioinformatics workflow, thus providing a limited framework for developing complex pipelines. Differently, RBP represents a new instrument, which expands the idea of Almugbel [28], providing a more flexible infrastructure allowing the bioinformatics community to spread their work under the guidance of rules, which guarantee inter-laboratory reproducibility and do not limit docker implementations to Bioconductor packages. Moreover the RBP project, differently by others projects i.e. snakemake and nextflow, is specifically designed for the R community.

The RBP workflows are designed to work on a single machine with multi-cores, which do not need to be necessary a high-end server [29]. In [29] we describe that RNAseq, miRNA-seq and ChIP-Seq workflows (Fig. 2) can be executed efficiently on a consumer computer equipped with Intel i7 CPU (8 threads), 250 Gb SSD disk and 32 Gb of RAM. Recently, with the implementation of the reference free aligner Salmon [22] the minimal RAM requirements dropped to 8 Gb. This make possible the execution of the workflows available in RBP nearly any modern laptop with Linux operating system. Of course, a high-end server allow an higher level of parallelization in the analysis of multiple samples. The advantage of a high-end server become also evident in case of the analysis of large datasets, e.g. whole genome variant calling or thousands of RNAseq experiments.

A future work will be to extend our project to deal with cluster and cloud architectures. Two possible directions will be investigated (i) to exploit the swarm mode provided by docker considering each service as a “single-shoot” service, and (ii) to provide an automatic translation of our workflow specified in R into an equivalent workflow specified in snakemake format or in nextflow format.

## Availability and requirements

**Project name**: Reproducible Bioinformatics Project.

**Project home page**: http://reproducible-bioinformatics.org

**Operating system:** UNIX-like.

**Programming language:** R.

**Other requirements:** docker version 17.05.0-ce or higher.

**License:** GPL.

## Abbreviations

ChIP-seq: Chromatin immuno precipitation sequencing
miRNA-seq: microRNA sequencing
OS: Operation system
PCA: Principal component analysis
PDX: Patient derived xenograft
RBP: Reproducible bioinformatics project
sncRNA: Human small non-conding RNA
SNVs: Single nucleotide variants
